# Nutritional and Pharmaceutical Applications of Under-Explored Knottin Peptide-Rich Phytomedicines

**DOI:** 10.3390/plants11233271

**Published:** 2022-11-28

**Authors:** Francis Alfred Attah, Bilqis Abiola Lawal, Abdulmalik Babatunde Yusuf, Oluwakorede Joshua Adedeji, Joy Temiloluwa Folahan, Kelvin Oluwafemi Akhigbe, Tithi Roy, Azeemat Adeola Lawal, Ngozi Blessing Ogah, Olufunke Esan Olorundare, Jean Christopher Chamcheu

**Affiliations:** 1 Department of Pharmacognosy and Drug Development, Faculty of Pharmaceutical Sciences, University of Ilorin, Ilorin 240272, Nigeria; 2 School of Basic Pharmaceutical and Toxicological Sciences, College of Pharmacy, University of Louisiana-Monroe, Monroe, LA 71209, USA; 3 Department of Biotechnology, Ebonyi State University, Abakaliki 480101, Nigeria; 4 Department of Pharmacology and Therapeutics, University of Ilorin, Ilorin 240272, Nigeria

**Keywords:** cystine knot, knottins, dietary phytomedicines, therapeutic knottins, food-grade knottins

## Abstract

Phytomedicines reportedly rich in cystine knot peptides (Knottins) are found in several global diets, food/herbal supplements and functional foods. However, their knottin peptide content has largely been unexplored, notably for their emerging dual potentials at both the food and medicine space. The nutritional roles, biological targets and mechanism(s) of activity of these knotted peptides are largely unknown. Meanwhile, knottins have recently been unveiled as emerging peptide therapeutics and nutraceuticals of primary choice due to their broad spectrum of bioactivity, hyper stability, selective toxicity, impressive selectivity for biomolecular targets, and their bioengineering applications. In addition to their potential dietary benefits, some knottins have displayed desirable limited toxicity to human erythrocytes. In an effort to appraise what has been accomplished, unveil knowledge gaps and explore the future prospects of knottins, an elaborate review of the nutritional and pharmaceutical application of phytomedicines rich in knottins was carried out. Herein, we provide comprehensive data on common dietary and therapeutic knottins, the majority of which are poorly investigated in many food-grade phytomedicines used in different cultures and localities. Findings from this review should stimulate scientific interest to unveil novel dietary knottins and knottin-rich nutraceutical peptide drug candidates/leads with potential for future clinical application.

## 1. Introduction

### Description, Distribution, Diversity, Abundance and Type of Knottin Peptides

An understanding of the role of plants as human–environmental co-tenants, which occupy the dual position of application as food and medicine, encouraged their domestication by man over 10,000 years ago [1]. Gradual domestication of plants over the years encouraged the careful selection of useful plants for nutritional purposes and to meet indigenous healthcare needs [2]. Such is the case of many plants including members of the grass family, legumes thought to have been domesticated over 2500 years ago in ancient India [3]. During this early human civilization in India and Africa, many of these plants currently occupying the interface of food and medicine [4] have been used by pastoralists to meet healthcare needs, nutritional requirements, feed farm animals or to improve the quality of polluted water [4]. The phytochemical content of plants informs their selection for nutritional or medicinal purposes. Toxic plants are often avoided except for well-informed applications such as for fishing, hunting etc. [5]. While the ubiquitously distributed primary plant metabolites including proteins, lipids and carbohydrates mainly serve nutritional purposes, the species-specific secondary compounds have been shown to produce pharmacologic or toxic activities [6]. However, scientific investigations have been focused more closely on non-peptidic secondary compounds such as alkaloids, phenolics and terpenes; biologically active peptidic plant metabolites have not received considerable attention until this decade [7]. The peptide family known as plant knottins (also called cystine knot peptides), which are distributed in plants growing more densely across the wetlands, are even more underexplored despite the therapeutic potential of these peptidic compounds [8]. Knottins are a structurally unique and functionally diverse class of disulphide stabilized peptides of low molecular weight whose architectural design has been naturally stabilized by an amazing cystine knot [9]. Despite the distribution of knottins in plants, their abundance, stability and diverse biological activity currently documented, these therapeutically interesting compounds appear to be among the least investigated plant constituents, particularly in plants making up important phytomedicines that have been considerably standardized and commercialized [8]. Very recently, limited attention of the relevant scientific community is focusing on investigating the peptidic content of phytomedicines used in various global ethnomedicines, particularly those applied in Western and Asian ethnomedicines [10,11,12,13].

**Figure 1:**
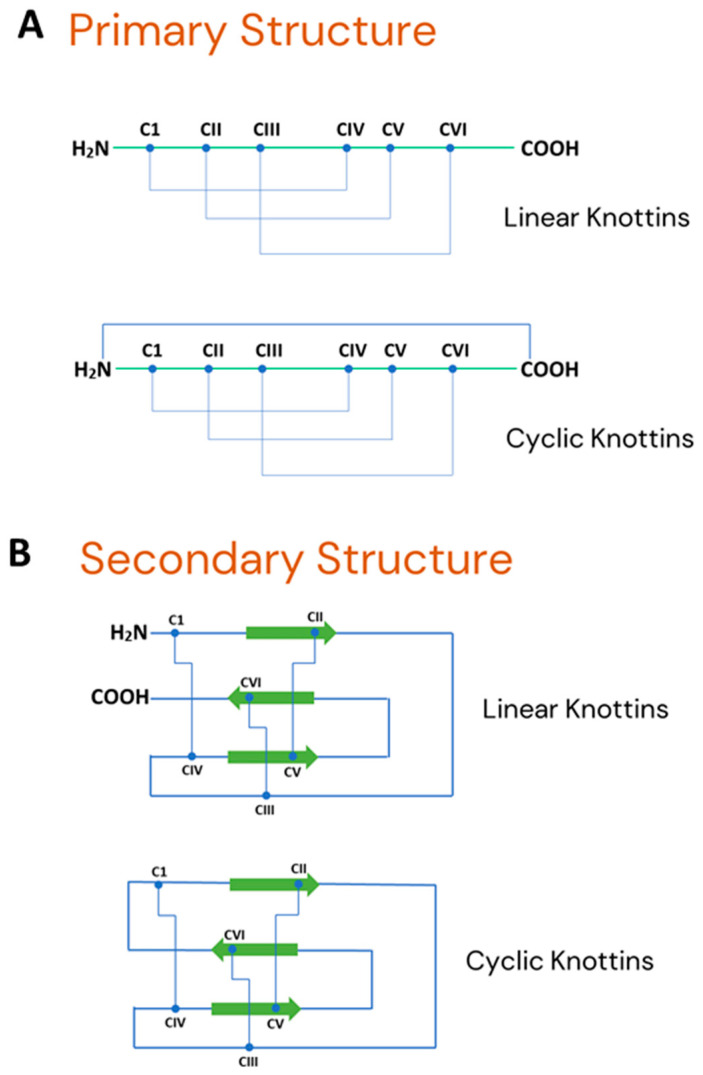
Representative primary and secondary structures of cystine knot peptides (knottins): (**A**) Primary structure showing diagnostic intercysteine connectivity as well as free N- and C-termini for linear knottins and connected termini without free amino and carboxyl termini expressed naturally by cyclic knottins; (**B**) Secondary structural backbone showing antiparallel sheet stabilized by a cystine knot. Strands are shown in orange and the six cysteine residues that form the cystine knot are labeled 1–6. While the disulphide bonds are known to be responsible for the extremely stable nature of knottins, the continuous circular configuration in cyclic knottins additionally makes the N- and C-termini unavailable for attacks by external digestive agents.

**Figure 2:**
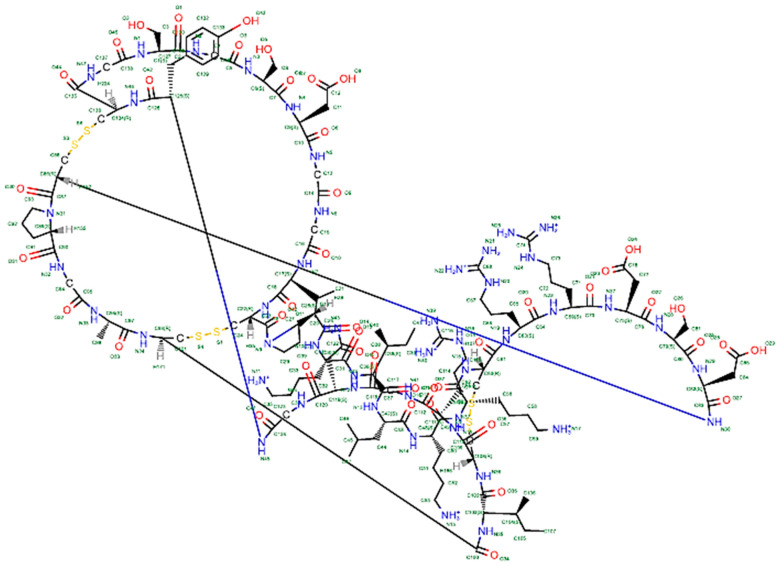
2D representation of a typical knottin peptide, a cyclic protease inhibitor (Trypsin inhibitor, MCoTI-II) isolated from the seeds of an important medicinal plant, *Momordica cochinchinensis* (Cucurbitaceae). This gene encoded cyclotide is abundantly expressed at the peptide level. The two nearly parallel disulfide bonds have been cross linked by a third one which passes through each of the two to form a knotted structural configuration that induces enormous stability in this bioactive knottin-like peptide. PDB:1HA9.

**Figure 3:**
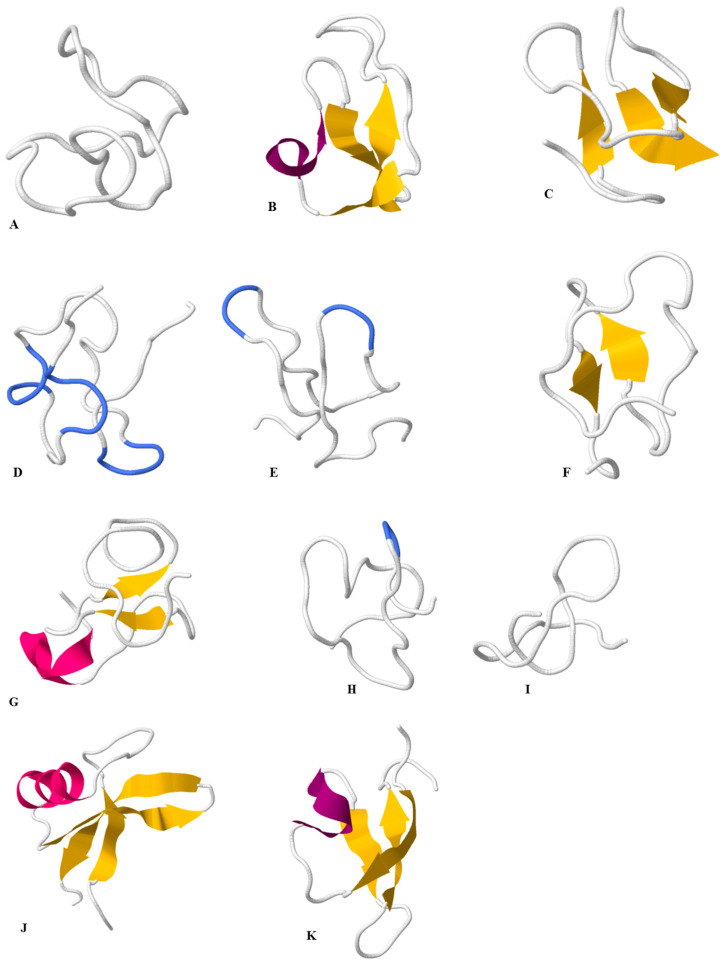
Molecular families of cystine knot peptides which include: (**A**) (PDB:1K48) Kalata B1 cyclotide from *Oldenlandia affinis*; (**B**) (PDB:1HA9) disulphide-rich protease inhibitor (squash trypsin inhibitor MCOTI-II from *Momordica cochinchinensis*); (**C**) (2MAU) alpha-amylase inhibitor wrightide R1 (wR1) peptide from *Wrightia religiosa*; (**D**) (PDB: PDB:6JIC), carboxypeptidase inhibitor from *Lycium barbarum*; (**E**) (PDB:5XBD) and (**F**) (PDB:2ML7) plant defensins, respectively, from *Pereskia bleo and Panax ginseng*; (**G**) (PDB: 5XDI) Vaccatide, vH1- an antifungal Glutamine-rich 8C-Hevein-like Peptide from *Vaccaria hispanica*; (**H**) (PDB: 5GSF) Roseltide rT1, a hydrolase inhibitor from *Hibiscus sabdariffa*; (**I**) (PDB: 5XIV) Beta-Ginkgotides from *Ginkgo biloba*; (**J**) (PDB: 2M8B) plant defensins AhPDF1 from *Arabidopsis halleri*; (**K**) (PDB: 1P8B) Pea albumin peptide (PA1b), insecticidal peptide characterized from *Pisum sativum*.

The term “knottin peptides” refers to a group of small protein molecules (typically < 6 kDa) that have three cysteine bonds (disulphide bonds), conformed rigidly to form a knot, which confers high thermal, molecular and chemical stability. The cystine-knot arrangement in the knottin family is most commonly C1−C-4, C-2−C-5, and C-3−C-6 [14] as demonstrated in Figure 1 (primary sequence) and Figure 2 (2D structure of a typical knottin peptide). These molecules tend to have side loops of amino acids with variable lengths that are tolerant to mutations, thus making knottins suitable candidates for drug design. The most notable molecular families (Figure 3) with this type of knot include cyclotides, disulphide-rich protease inhibitors, which include trypsin, carboxypeptidase and α-amylase inhibitors, Pea albumin peptide (PA1b), some plant defensins and some hevein-like peptides [15,16]. In terms of pharmacological activities, knottins have been observed to interact with plasma membranes and ion channels. This is responsible for their antimicrobial activity, which is observed in their respective botanical sources as they are known to be pathogen resistant [17]. In addition to this, knottins have been found to possess antihelminthic, antimalarial, anti-HIV, uterotonic, insecticidal, anti-tumor and anti-fungal activities [9]. With respect to their molecular structures, plant knottins are either cyclic in nature, predominantly known as cyclotides, or non-cyclic in nature, which include the other linear members of the family [17].

Cyclotides are a family of cyclized knottins which are subdivided into three subfamilies—bracelet, mobius and trypsin inhibitors [17]. The representative of this family is the KalataB1, a cyclotide obtained from *Oldenlandia affinis*, whose discovery in 1970 spurred the research into protein drugs with circular knotted configuration [18]. It was discovered because of its uterotonic activity; however, since then findings have revealed it possesses a plethora of pharmacological activities such as antimicrobial, insecticidal, immunosuppressive, hemolytic and uterotonic activity [9,18,19]. Further investigations are underway and a variant, T20K, has been identified as a candidate for treating multiple sclerosis [20]. Cyclotides are distributed mostly in the Rubiaceae and Violaceae families, but can also be found in Solanaceae, Cucurbitaceae and Fabaceae families [8,16]. Over the years various cyclotides have been isolated and their activities elucidated, such as Circulin A and B, isolated from *Chassalia parvifolia*, which were discovered to have anti-HIV properties and cycloviolacin O2, isolated from *Viola odorata*, which is cytotoxic and being investigated for treating cancer [8].

Unlike cyclotides, hevein-like peptides are linear and simply fall under a class of peptides, known as antimicrobial peptides (AMPs), which are distributed wholly in plants and are known to confer resistance to phytopathogens, exclusively fungi. This was elucidated to be due to their ability to specifically bind to chitin, an essential part of the fungal cell wall. Some identified hevein-like peptides, like Pn-AMP1 and Pn-AMP2 isolated from Morning Glory (*Pharbitis nil*), have activity against fungi possessing chitin and even fungi without chitin in their cell wall. Others have also shown activity against Gram-positive and Gram-negative bacteria such as Fa-AMP2, isolated from Buckwheat (*Fagopyrum esculentum*), and AvesinA, isolated from Oats, at micromolar concentrations [21]. Hevein-like peptides are named as such because their structures resemble Hevein, the first member of the family isolated from *Hevea brasiliensis*, and to date, only 20 members have been found in angiosperms. Although, recent findings have shown they may be present in gymnosperms with the discovery of ginkgotides, isolated from *Ginkgo biloba*, which also possess similar antifungal activity [22]. Subsequently, it is clear that the knottin peptides hold strong promise as drug molecules, drug scaffolds and intracellular drug delivery systems [19].

Findings from these literature reports strongly emphasize the therapeutic roles of knottins in the overall bioactivity of these standardized indigenous phytomedicines. However, there is a lack of a comprehensive review of this documented work appraising what has been accomplished, unveiling the huge knowledge gaps and shining the light on the future prospects of knottins at the interface of food and medicine. Herein, we aim to provide a comprehensive review on the nutritional and pharmaceutical potentials of the poorly investigated knottins-rich phytomedicines used across the globe in order to trigger scientific interest for further in-depth investigation, particularly in regions where they are least investigated. Findings from this review could drive the renewal of scientific interest in the discovery of novel knottin peptide drug candidates, and thus increase the potential for their clinical application.

## 2. Applications of Knottin-Rich Peptides

### 2.1. Nutritional Applications of Knottin-Rich Phytomedicines

This family of chemically identical knottins, also known as cystine-knot miniproteins, does have a diversified amino acid composition and varied biochemical roles [23]. The role of “edible” cystine knot peptides (knottins) as innovative food peptides is largely yet to receive scientific assessment for their nutritional and functional roles. The dietary quality of food-derived knottins has not been documented. Meanwhile, knottins have been identified and characterized from several food/herbal recipes important in human diets [24]. Common examples of cystine knot peptides and Knottin peptide-rich plants commonly taken as food have been summarized in Table 1; they include cyclotides from *Clitoria ternatea* [25], PA1b peptides from *Pisum sativum* [26], knottin-like protease inhibitors from *Beta vulgaris* [13], knottin-type Roseltides from *Hibiscus sabdariffa* [27], Morintides from *Moringa oleifera* [28], knottin-type antimicrobial peptides from *Amaranthus* spp. [12,29] and antibacterial knottin-like peptides from *Ghost pepper* [30] and other *Capsicum* species recently reviewed by Oliveira and colleagues [31]. The contributory nutritional role of these knottins in the diets requires deliberate scientific attention. For instance, knottin peptides, although considerably stable to enzymatic attack, when hydrolyzed in the gut may act as a good source of dietary amino acids while the unhydrolysed stable peptide has the potential to trigger changes in the gut microbiota [32] owing to their broad bioactivity, antimicrobial activity in particular [33]. Medicinal plants and phytomedicines rich in knottin-like peptides commonly taken as food appear to be good sources of essential amino acids and low molecular weight peptides [34] needed for the proper physiological functioning and prevention of several metabolic diseases [35]. These essential and other types of important dietary amino acids have thus been classified as functional amino acids [35].

*Clitoria ternatea* (Darwin Pea): The plant, known as the butterfly pea, has been grown for many years as a fodder plant, but recently it is being explored for its dietary applications as well as potential uses in medicine and agriculture [11]. *C. ternatea* is a plant with flowers, having an abundance of anthocyanins, which predisposes its use as a food coloring agent and additive to improve aesthetic appeal [11]. Locally, its flowers have been used to color a popular Kelantan dish known as the *nasi kebaru* blue, which can be eaten with grilled chicken, fried fish coated with flour, fish crackers, salted egg and other local herbs [36] The plant could serve as a source of essential amino acids and protein whose content has been reported to be around 40% of mean weight [37]. The novel discovery of abundant ultrastable circular knottins (cyclotides) in *C. ternatea* opened the doors to potentially greater innovation that could unveil the dietary roles of these cystine knot peptides. One of such cyclotide-rich extract, Sero-X, was approved for use in Australia in 2017 as an eco-friendly biopesticide with an overall advantage of improving agricultural practices [11].

*Pisum sativum*: The Garden Pea is a staple that makes up several diets worldwide due to its high protein and amino acid content, within a range of 21–30% of mean weight [38]. This nutritional content makes the plant a valuable food source that has even been proposed to meet the dietary needs of about 800–900 million undernourished people worldwide [39]. It is now considered as an add-on for cereals to boost their nutritional value and as a substitute for animal proteins [38]. The garden pea is also rich in phenolics, natural antioxidants which would contribute to normal general wellbeing [39]. The Garden Pea has been reported to be a rich source of cysteine-rich knotted peptides known as Pea Albumin 1 subunit b peptides (PA1b), a highly selective entomotoxin abundantly expressed in the edible seeds of the pea plant [26]. *P. sativum* has also been reported to produce peptide constituents that confer antimicrobial resistance; Psd1 and Psd2, isolated from *P. sativum*, are some plant defensins which have already been proven to possess some antifungal activity [40]. However, the nutritional role of these knottin peptides expressed in the plant and related species like *Cajanus cajan*, which potentially express cysteine knot peptides, await scientific investigation.

*Hibiscus Sabdariffa*: The calyces of *Hibiscus Sabdariffa* are a source of proteins with 7.51% protein on a dry weight basis [41]. For a very long time, crude extract and calyx powder has been employed as a food source and nutraceuticals in most parts of Africa and Asia [42]. Most nutritional and pharmacological activity has been linked to bioactive constituents such as organic acids, anthocyanins, polyphenol and flavonoids. Only recently, roseltides, a knottin-rich neutrophil elastase inhibitor, were discovered [43]. Necessary scientific attention has not been placed on the contribution of these ultra-stable knottins to food and medicines. Moreover, it is yet to be investigated the percentage of the proteins that are knottin-rich and how they could contribute to nutritional and pharmacological benefits. Though knottins are stable in the gastrointestinal tract, protease can break down the knottin-rich peptide after an hour. These enzymes could help break down knottins into the individual amino acid residue, which could then be absorbed, and this contributes to its nutritional benefit. On the other hand, the undigested knottins after absorption tend to retain the conserved cysteine-knot motif and could become an excellent chemical entity contributing to the reported pharmacological activity of the crude extract and powdered calyx. However, while that study is yet to be investigated, it deserves research attention.

*Beta Vulgaris* (beetroot) has been used industrially as a source of sugar and traditional medicine in treating constipation, decreased libido, gut and joint pain and dandruff [44]. The nutritional content is due to a high protein constituent of approximately 13.23 mg/100 g [45]. Retzl and colleagues [13] recently identified a novel knottin-rich inhibitor peptide, bevuTI-I, from beetroot and showed that it belongs to the trypsin inhibitors family with a potential therapeutical advantage in the management of autoimmune diseases and cancer. Beetroot is majorly grown for food uses (pickles, salad, juice) rather than for sugar production. The extract has been employed in the management of cancer and coronary heart diseases due to its anti-inflammatory effect [46]. Further studies are needed to investigate the potential contributions of the knottin-rich peptide to the nutritional and pharmacological benefit derivable from the plant, as nature is not wasteful in natural product biosynthesis.

*Amaranthus* spp., also known as Amaranths, constitute a group of edible plants known as Pseudo-cereals which are consumed not only for their food use but also for their medicinal benefits [47]. This dual character as a food and drug serves as the basis for its economic importance, even in poor countries. *Amaranthus* constitutes a cosmopolitan genus with common species such as *Amaranthus hypochondriacus*, *Amaranthus cruentus*, *Amaranthus hybridus*, *Amaranthus caudatus* and *Amaranthus tricolor* [48]. While some of these species are known for insecticidal activity, some are known for an antimicrobial property. For example, *Amaranthus caudatus* contains known characterized Knottin peptides which are very effective in inhibiting the growth of fungi and Gram-positive bacteria [49]. The seeds of *A. hypochondriacus* contain α-amylase inhibitors which are effective for their insecticidal property against the larvae of various insects such as *Prostephanus runcates*, and *Tenebrio molitor*. The amaranth alpha-amylase inhibitor’s (AAI) three-dimensional structure adopts an abcabc knottin fold [50]. Mammalian amylases are not affected by the α-amylase inhibitors due to selective activity [49]. Amaranths are useful nutraceuticals across the different species due to the variety of proteins they contain such as Ar-AMP from *Amaranthus retroflexus*, which is an effective antifungal against pathogens like A. consortiale, *B. cinerea*, etc. Ay-AMP from A. hypochondriacus which is also a useful antifungal against *C. albicans*, *G. candidum*, *A. alternate*, *F. solani*, etc. [51]. Studies have clearly summarized the wide importance of the various species under the Genus *Amaranthus* with properties across insecticidal activity, fungicidal activity and bactericidal activity. The presence of knottin-rich peptides in the seeds of this plant underlies its use as a Nutraceutical and its usefulness in Phytomedical applications.

*Capsicum species*, an important dietary ingredient used globally as a spice to add flavors to dishes, has been documented as a rich source of cystine knot antimicrobial peptides [52] which have not been elaborately studied. In Nigeria, particularly south-west Nigeria, *Capsicum species* are highly valued in diets as the plant species have strong historical claims of disease prevention and health restoration during infections and viral pandemic (personal communication). However, the complementarity and nutritional roles of these peptides in Capsicum-rich diets await scientific investigation.

*Moringa oleifera* Lam. (Moringaceae) is a tropical plant which has been domesticated in various parts of Africa and Asia where it is consumed as a nutrient-endowed food and medicine [4]. The reported antimicrobial knottin peptides [28] MO1 and MO2 and other water clarifying polypeptides have attracted scientific attention [53]. However, the nutritional relevance of these knottin-like peptides in maintaining gut integrity, improving food digestibility as well as other physiological functions have not been explored. However, these peptides are constantly released into water during water clarification treatment or when Moringa leaf is taken as tea. For instance, in northern Nigeria, the knottin peptide-rich leaf has a history of use as a traditional food/salad by the Hausa-Fulani ethnic nationality (personal communication).

Proteins and peptides from many sources have been studied extensively to develop important foods [54]. When testing protein-rich products, emulsification is a vital step in the process. Emulsifying properties of food bioactive peptides byproduct depend on their amphiphilic nature [55]. Emulsifying properties have been observed in plant-derived active peptides such as in potato, flaxseed, and soybean [56]. The potential emulsifying property of knottin peptides isolated from phytomedicines used as food additives have not been investigated.

Functional diets based on the usage of phytomedicines rich in knottin peptides are widely used across the continents but are under investigated. Knottin peptide-rich phytomedicines have proven to be particularly beneficial as food additives, in beverages and many other yet-to-be validated nutritional applications including as sources of essential amino acids. For example, these peptides may hold some promise as sugar substitutes or some color stabilizers, a thickener or an anti-caking factor. It is not known if knottin peptides have the potential to enhance the flavor or influence the acidity of food. By altering the water and oil retention, colloidal stability, viscosity and foam production in the end product, knottin peptides may improve food quality [57]. However, this informed guess begs for scientific investigation.

**Table 1:** Reported knottin-rich phytomedicines used in various parts of the globe as both food and medicine (nutraceuticals) and their botanical sources.

Phytomedicine/ Botanical Source/Country	Knottins Present	Nutraceutical Applications	Scientific Evidence	References
Ewa/ *Clitoria ternatea* L. Indonesia	Cyclotides, e.g., Cter M	*C. ternatea* flowers are used as a natural coloring for food and beverage. It helps to improve food quality by affecting the water and oil retention capacity, colloidal stability, viscosity, and foam generation in the finished product. It possesses anti-osteoporotic, antihypertensive activity and reduces cardiovascular complications.	In silico, in vitro and in vivo	[58,59]
Ipecac/ *Carapichea ipecacuanha* (Brot.) L. Andersson Brazil	Caripe Cyclotides (Caripe 1–13)	It helps to minimize the cooking/frying loss and shrinkage and enhances foaming stability in chicken sausages. Amoebic dysentery, cough, whooping cough and bronchitis can be treated using root extracts and powdered leaf of Carapichea. *C. ipecacuanha* contains emetine and cephaeline, to induce vomiting. It helps to reduce the risk of certain chronic diseases such as leukemia. The natural action of the bioactive compounds protects the biological attributes such as anti-aging, anti-inflammatory, anti-viral, anti-microbial, and anti-cancer properties.		[60,61,62,63]
Beetroot/ *Beta vulgaris* (L.) Arcang Mediterranean Europe and North America	bevuTi-I	Beet juices, peels and pomaces is a plausible source of iron and helps in treating malnutrition and iron deficiency anemia. Beetroot extracts have a considerable number of antioxidants which are used in heavy metal toxicity and cancer chemoprevention. Studies have reported the use of beetroot supplementation as an hypoglycaemic and antihypertensive agent. Beetroot is a rich source of calcium, consumption of 100 g of beets powdered leaves meets daily recommendations as prescribed by FDA (1000 mg/day). Beetroot is also a rich source of Vitamin C, helps in immune function and is a great source of folate (vitamin B9) which helps cells and tissues to grow and function. Nitric oxide in beets acts as a vasodilator, thereby reducing blood pressure. It is used as a natural food colorant and labeled as E-162. The peptide present in beetroot is a promising drug against neurodegenerative disorders such as Alzheimer and Multiple Sclerosis.	In silico, in vitro and in vivo (clinical trials)	[13,64,65,66]
Oruwo/ *Morinda Lucida* Benth. Senegal to Sudan and southward to Angola and Zambia	Unknown cysteine stabilized peptides (CSP)	The root, stem bark and leaves of *M. Lucida has* been used traditionally in the treatment of malaria, sickle cell disease, hypertension, diabetes and other diseases. The antioxidants Vitamin A and E in *M. lucida are* used in battling degenerative disease like atherosclerosis. Vitamin K can be found in high quantities in the leaves of *M. Lucida* and helps in building strong bones. Its dietary fiber plays an important role in satisfying human needs for energy The bitter tasting roots are used as flavoring for food and alcohol. The wide range of enzyme and vitamins present in *Morinda lucida* makes it useful in treatment of different disease conditions.	In vitro, in vivo and in silico	[67]
Roselle/ *Hibiscus sabdariffa* L. Angola	Roseltide	*H. sabdariffa* is used as food coloring in food industries by incorporating their calyces. Extractions or infusions of dried calyces of zobo flower are used as tea (beverage) to lower body temperature, blood pressure, blood sugar and cholesterol. The leaves and calyces are eaten with pulverized peanuts in Africa as a side dish. It is used a good source of vitamin C and iron It is used with lemon balm and St John’s wort to treat restlessness and poor onset of sleep. Hibiscus tea is used as a mild laxative.	In silico, in vitro and in vivo (clinical trials)	[68]
Pea/ *Pisum sativum* L. Middle East specifically to Turkey and Iraq	PA1b (Pea Albumin 1 Subunit b)	The major components of peas are protein, starch and fiber which help in breaking down carbohydrates, hence are beneficial in the prevention and management of type-2 diabetes. Coumestrol in peas helps to lower the risk of stomach cancer. Addition of peas to food in combination with inulin fiber supplement greatly increases bowel movement frequency. It is a functional ingredient in vegan foods and dried seeds of pea are used as staple foods. Eating green peas regularly helps to lower blood pressure and blood cholesterol level due to the mineral content (Mg, K and Ca) present in them.	In silico, in vitro and in vivo	[39,69]
YinXing/ *Ginkgo biloba* L. China	β-Ginkgotides	*Ginkgo*-steamed eggs and *Ginkgo*-fried chicken are popular treats in Southeast Asia. Moreover, *G. biloba nuts* are used as a side dish in Japan. *G. biloba* seeds are largely encouraged to be used as nutraceuticals for prevention of neurodegenerative diseases. *G. biloba* seeds entail a higher percentage of vitamin C, carbohydrates, riboflavin, proteins and other nutrients. *G. biloba* seeds are incorporated in alcohols, drinks, glazed vegetables, cakes, sweets and other delicacies.	In silico, in vitro and in vivo (clinical trials)	[70]
Korean ginseng/ *Panax ginseng* C.A.Mey China	Ginsenosides	Ginseng tea, the decoction of ginseng roots is a natural home remedy to manage hypertension. It is used in weight loss management. Ginsenosides present in ginseng are chemically similar to female steroidal hormones, hence the tea helps to restore hormonal balance in women. Ginseng is used as a spice to promote skin health due to the presence of free radical fighting antioxidants. Ginseng tea and wine improve sexual health in men. Use of ginseng improves erectile function, sexual desire and intercourse satisfaction. Studies have shown the efficacy of *P. ginseng* in Chronic Fatigue Syndrome, normal fatigue, cancer related fatigue and postoperative fatigue syndrome.	In silico, in vitro and in vivo (clinical trials)	[71,72,73,74,75,76]

### 2.2. Pharmaceutical Applications of Phytomedicines Rich in Knottins

Organic compounds and protein biologics have a lot in common, but knottins have evolved as intriguing prospects for therapeutic development because of their ability to adhere to clinical substrates with strong affinity and specificity [23]. Knottins also show promise in challenging pharmaceutical research goals, nutraceutical uses such as oral distribution, and the capacity to access cellular therapeutic targets due to their extraordinarily high tenacity and unique structural properties [54].

Degree of Evidence: the pharmaceutical applications of the underexplored knottins-rich peptides have been presented based on scientific evidence documented to support the pharmacological action. As a result, to unveil the extent of scientific evidence supporting the reported knottins, the following classifications have been used:

Class I—At least one clinical investigation has validated the evidence (most significant degree of scientific evidence).

Class II—An in vivo experiment was used to validate a documented pharmacological activity.

Class III—In vitro experiments provide evidence for the reported pharmacological activity.

Class IV—Evidence supported by in silico study or extrapolated from the crude extract.

In this review, these established evidence levels are denoted by square brackets, such as [Class I] for claims obtained from at least one clinical investigation [77].

### 2.3. Antimicrobial

Among the cyclotides identified from coffee plants are Kalata B1, Circulin A, Circulin B, and Cyclopsychotride A, which were the first knottin-rich peptides with antibacterial properties to be described [78,79]. More cyclotides with antibacterial activity were subsequently discovered (Table 2).

Hedyotide B1, a cyclotide isolated from the aerial part of *Hedyotis biflora*, showed potent activities against Escherichia coli and Streptococcus salivarius (MIC 3.4–5.9 μM), while no activities were reported for Staphylococcus aureus and Staphylococcus epidermidis (Class III) [80]. However, the same plant’s root-derived hedyotides B10 and B11 had more substantial effects against *S. epidermidis* and *S. aureus* (MIC 1.5–2.2 M) [81]. The trypsin inhibitor cyclotides (MCoT-I and MCoT-II) from *Momordica cochinchinensis* are devoid of activity against all tested organisms, even at maximum concentration of 160 μM [82]. When five knottin-rich peptides (CyO2, Kalata B1, Kalata B2, Vaby A, and Vaby B) were compared for their antibacterial activity, it was found that CyO2 was the most effective against Gram-negative bacteria (*S. enterica* MIC 8.75 μM and *E. coli* MIC 2.2 μM). In contrast, Gram-positive bacteria are more resistant to treatment (*S. aureus* MIC > 50 μM) (Class III). However, Kalata B1 was devoid of activity even at peptides concentration >100 μM, while Kalata B2 showed moderate activity (MIC 35 μM) against all tested organisms (Table 2).

Recent studies [82] showed that the cycloviolacin group of bracelet cyclotides (CyO2, CyO3 and CyO-19) exhibited broad spectrum activity, with significant activity against *S. aureus*. Similar to this, CyO2 and Kalata B2 were tested for their anti-staphylococcal efficacy against *S. aureus*. CyO2 showed the highest in vitro activity (MIC 25 μM), whereas Kalata B2 displayed somewhat lesser potency (MIC 50 μM). Considering the broad spectrum of activity and potency of CyO2, further studies are needed to demonstrate its antimicrobial activity against microorganisms resistant to commonly available antibiotics. This could open the floor for developing knottin-rich antimicrobial peptides with potential clinical application. The result was consistent with the first in vivo experiment conducted on a surgical *S. aureus* wound infection mouse model (Class II) [83].

Cyclotides from *O. affinis* displayed considerably different antimicrobial properties. Kalata B1, a popular uterotonic agent, was inactive against *E. coli*, whereas moderate activity was reported for *P. aeruginosa*. On the other hand, Kalata B7 displayed potent activity against *P. aeruginosa* (Class III) [78].

Generally, *P. aeruginosa*, *E. coli*, and *S. aureus* were the most vulnerable to cyclotide-based antibacterial activity [82]. Experiments have shown that the antimicrobial properties of cyclotides depend on various salt concentrations. In general, low salt concentration resulted in increased activity while a high salt concentration decreases or abolishes activities. Only the antimicrobial cyclotides circulin B, cyclopsychotride A, and cliotide T15 retained antimicrobial activities, both in low and medium salt concentration. Hence, they are promising lead compounds that can be explored for peptide-based antimicrobial agents [25]. In addition, ref. [84] isolated and characterized potent and selective bactericidal peptide *Echinopsis pachanoi* antimicrobial peptide (Ep-AMP1) with extremely low cytotoxicity. This peptide demonstrated significant activity against Gram-positive bacteria, weak activity against Gram-negative bacteria, and no activity against *C. albicans* even at concentrations exceeding 160 M. (Table 2) [84].

Recently, Ref. [85] synthesized cyclotide VarcA previously identified from *Viola arvensis* and demonstrated their antimicrobial activity against bacterial infection in aquaculture. Similar, cyclotides isolated from *Sambucus nigra* L. flowers demonstrated effectiveness against several pathogenic Gram-negative bacteria that threaten fisheries. Though the peptide is yet to be characterized, their proposed mode of action results from peptide interaction with the lipid membranes of the bacteria resulting in membrane disruption, leakage of cellular content and ultimate death of the pathogenic organism [86].

### 2.4. Anticancer

Knottins-rich peptides present a good protein scaffold that could be explored for potential application in cancer drug treatment and delivery [87]. The exceptional thermal, chemical and enzymatic stability, coupled with a distinct structural-activity-relationship (SAR) which is amenable to chemical modification, present a unique field that could be exploited to develop promising drugs for the management of cancer. This class of peptide not only possesses anticancer properties but also the ability to serve as a carrier in cancer drug delivery as peptides-drug conjugates, thereby facilitating its entry into the blood–brain barrier, improving target affinity and specificity, and also the ability to bypass drug resistance mechanisms associated with conventional anticancer drugs [88,89].

Cytotoxic properties of knottin-rich peptides have been thoroughly documented, particularly for cyclotides; Grover et al. [89]. compared this activity and found that around 74% of the cyclic knottins demonstrated action at a relatively low micromolar concentration (7 M), confirming their efficacy against the tested cancer cell line. CyO2 isolated from various *Viola* spp., including *Viola odorata*, appear to be the most potent, promising and well-studied. In addition to being one of the most promising antimicrobial peptides, their high cytotoxicity presents an exciting opportunity for potential development as antitumor therapeutics. However, an in-depth understanding of possible interaction with the DNA of cancer cells needs to be established. Three novel cyclotides (Psyle A, C, and E) from *Psychotria leptothyrsa* (Rubiaceae) were compared to CyO2 in terms of their cytotoxicity, and it was found that they are both effective against breast cancer cells [90]. Additionally, they reported that they could create pores in drug-resistant breast cancer, which facilitates the uptake of the chemotherapeutic drug Doxorubicin. The synergistic effect of this combination requires further investigation to determine toxicity and efficacy, with the potential development as either monotherapy or fixed combinational therapy with an existing chemotherapeutic agent.

Among the cyclotides isolated from *Hedyotis biflora Hornem*. (Accepted name: *Leptopetalum biflorum* (L.) *Neupane* and *N.Wikstr*.), Hedyotide B7 is the most potent in both in vitro and in vivo experiments against pancreatic cell lines (Table 2) [91]. However, when tested using human prostate cancer xenografts (Class II and III), the *H. diffusa* cyclotides derived from the root and leaves of *H. diffusa* showed potent cytotoxic activity.

Though the crude extract *H. diffusa* has been approved clinically for the management of colorectal cancer, its cyclotides are yet to be investigated for this activity (Class I) [92]. Hemolytic activity is often a significant setback for the potential development of knottin-rich chemotherapeutic agents; however, lysine-rich cyclotides from *M. latifolius* displayed limited hemolytic activity even at a very high peptide concentration (Table 2) [93]. Hence, they are promising candidates for anticancer drug delivery/design since toxicity to erythrocytes is a major setback in some toxic knottin-rich peptides.

Cliotide T1 showed the most potent cytotoxicity in HeLa cell line among the twelve cyclotides isolated from *C. ternata* with IC_50_—0.6 µM [80,82]; Vigno 5 from *Viola ignobilis* recently demonstrated dose-dependent cytotoxicity against Hela cells by apoptosis linked to the release of cytochrome C and increased activity of caspase-9 and -3 in Hela cells [94].

Numerous pharmacological properties of *Hibiscus sabdariffa’s* crude extract have been scientifically demonstrated, including the ability to lower blood pressure, improve memory, prevent cancer, reduce inflammation, and act as an antioxidant [42]. However, knottin-rich extracts have not yet been studied for these properties. Recently, Loo et al. [43] identified Roseltides (rT1–rT8), a new knottin-rich human neutrophil elastase inhibitor with promising therapeutic relevance, in neutrophil elastase-related disorders. In addition, rT1 and rT7 is a cell-penetrating peptide that selectively targets the mitochondria and enhances ATP production, which could present a therapeutic option in managing disorders associated with ageing, such as cancer [27]. Further studies are needed to investigate these peptides’ contribution to the pharmacology of the crude extract. Exploring how these peptides contribute to the neutraceutical properties of the extract after being subjected to high-temperature conditions during production will be intriguing, given their outstanding thermal and enzymatic stability.

The cysteine-knot Metallo carboxypeptidase inhibitors have been reported for *Solanum lycopersicum*, *Solanum tuberosum*, *Nicotiana tabacum* and *Hyoscyamus niger*, with various interesting yet unexploited pharmaceutical applications (Table 2). The tomato cysteine-knot mini-proteins (TCMP-2) have potent antiangiogenic activity via modulating vascular endothelial growth factor receptors [95]. Therefore, tomato consumption may help prevent various types of cancer and vascular disease due to the existence of TCMP-2 and other secondary metabolites with strong antioxidant properties [96].

**Figure 4:**
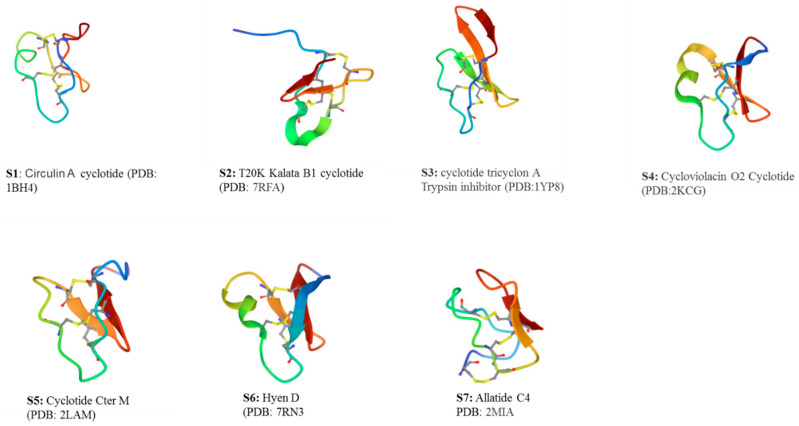
Three dimensional structures of some selected cystine knot cyclotides from botanical sources: (**S1**)—Antimicrobial cyclotide; (**S2**)—In clinical trials for multiple sclerosis; (**S3**)—Antifungal cyclotide; (**S4**)—Antibacterial cyclotide; (**S5**)—Anti-inflammatory cyclotide; (**S6**)—Anticancer cyclotide; (**S7**)—Antidiabetic cyclotide. The circular configuration complements the knotted structural core making knottin-like cyclotides the foremost peptide molecules for application in biotechnology, drug discovery and in agriculture.

In general, knottin-rich peptides possess potent cytotoxic properties, good cell penetration and a diverse SAR that could be modified for the development of the ideal chemotherapeutic drug candidate in the search for anticancer agents. For instance, the analysis of hyen D cyclotide (Figure 4S6) characterized from *Hybanthus enneaspermus* (Linn) has shown the functionally and structurally critical residues important for membrane binding and cytotoxicity [97].

### 2.5. Antiviral

Knottin-rich peptides, particularly cyclotides have been extensively investigated for their activity against viruses that cause diseases in humans, including the human immunodeficiency virus (HIV), the H1N1 influenza virus, and dengue (DENV) [98].

Circulin A and B (Figure 4) are the first group of cyclotides discovered to possess antiviral activity against HIV and were identified from the crude extract of *Chassalia Parvifolia* [99]. The proposed antiviral mechanism of action of the prototypical Kalata B1 is by interaction with the viral particles before entry, thereby preventing its fusion into the host cell membrane. By the early 2000s, numerous knottin-rich peptides had been identified with significant activity against HIV [100,101,102]. The prototypical Kalata B1 also showed strong activity against HIV by destroying the viral particles prior to entry, and also inhibiting fusion of the virus to host cell membrane [103,104,105]. Similarly, a kalata B1-inspired peptide produced from the amino acid modification of Kalata B1 presents significant activity against dengue virus (Class III) [106].

Alstotides and Allotides, botanical cystine knot α-amylase inhibitors, respectively, from *Alstonia scholaris* (Apocynaceae) and *Allamanda carthatica* L. (Apocynaceae), Produced α-amylase inhibition, while the Alstotides additionally showed significant membrane permeability and early phase antiviral activity against infection of bronchitis virus and Dengue infection (DENV2). For clarity of name, the solution structure of Allatide C4 (Figure 4S7) is an example of a characterized Allotide from *Allamanda carthatica*. These groups of knottin-rich peptides were shown to be highly stable in the metabolic environment; they selectively target the viral cell membranes and are non-cytotoxic even at peptides concentration up to 100 μM [107].

Anti-HIV activity has also been described for Cycloviolacin (O2, O13, O14, O24, Y1, Y4 and Y5) isolated from various *Viola* spp. Cycloviolacin Y5 is the most potent of the cyclotides isolated from *Viola philippica Cav*. while cycloviolacin Y1 is the least potent [108]. Gerlach et al. [90] showed that combining protease inhibitors (Saquinavir and Nelfinavir) with CyO2 increased their antiviral activity. This peptide accelerates pore formation in HIV-infected T-cells and monocytes, thereby increasing the absorption of the antiviral drugs. The antiviral mechanism of action of cyclotides is similar to that of antibacterial, mainly through interaction with the cell membrane resulting in cell shrinkage, leakage of cellular content and subsequent death of the pathogenic organism [18]. In the case of enveloped viruses such as HIV, these peptides destroy viral particles by disrupting the lipid membrane and thereby preventing its fusion into the host cell [105].

Despite the promise knottin-rich peptides have shown, before they are employed as antiviral agents, there is a need to assess their hemolytic effect on erythrocyte and cytotoxicity to human cells. Interestingly, these groups of peptides are amenable to structural modification to optimize their activity, improve pharmacokinetics profile and reduce toxicity.

### 2.6. Antifungal

The knottin-rich peptides displayed potent antifungal activity against pathogenic fungal species from plants and animals Table 2). The first reported antifungal activity from four knottin-like cyclotides (kalata B1, circulin A, circulin B, cyclopsychotride A—Figure 3 and Figure 4) were reported against *Candida kefyr*, *C. tropicalis* and *C. albicans* under different salt conditions. Under low salt conditions the four cyclotides showed moderate activity against *C. kefyr* and *C. tropicalis*, whereas no activity was reported for *C. albicans* [78]. Cycloviolacin O2, cycloviolacin O3 and cycloviolacin O19 showed potent activity against *C. albicans* with 99% activity seen at Minimum Fungicidal Concentration (MFC) of 10 µM (Class III) [109]. The same studies also reported the MFC of 40 µM for tricyclon A against *C. albicans* and low fungicidal activity was reported for cliotides (cter B, cter E, cter G) and kalata cyclotides (KB1, KB2, KB7 and KB13) [109].

Apart from their activity against fungal infection in humans, knottin-rich peptides also possess considerable activity against fungal infection in plants. It is unsurprising since this class of peptides primarily serve as defensive ‘molecular machines’ in plants. Slazak et al. [109] demonstrated that the antifungal activity of knottin-like cyclotides is similar to that of bacteria by selectively targeting and disrupting membranes containing phosphatidylethanolamine. Recently, the cycloviolacin (O2, O3, O8 and O19) are under scientific investigation as potential bio-fungicide against Fusarium head blight in crops [109,110].

The hevein-like, knottin-rich peptide Ginkgotides (gB1–gB11) and Vaccatide from *Ginkgo biloba* and *Vaccaria hispanica*, respectively, also showed potent activity against phyto-pathogenic fungi with an IC_50_ value within micromolar concentration (Table 2) [22]. This class of extremely stable knottin-rich peptide presents potential, but are currently underexploited as potential bio-fungicides and orally active and metabolically stable peptide-based therapeutics.

Plant defensin are a superfamily of knottin-rich antimicrobial peptide with cis configuration which majorly function as component of plant innate immune system This class of peptides confers protection to plants against pathogenic fungal and bacterial infection, mainly through membrane permeabilisation, generation of free radicals, specific lipid interaction, and initiation of cell wall stress and cellular leakage [111]. In addition, this class of peptide is involved in the development of metal tolerance and possesses inhibitory activity against α-amylase and trypsin. For instance, *Arabidopsis thaliana* Plant Defensin Type 1.1 (AtPDF1.1) is a secreted protein that can chelate apoplastic iron, and it presents a strong antibacterial activity against necrotrophic bacterium (*Pectobacterium carotovorum*) via an iron-deficiency defense response [112]. The plant defensin RBAFP isolated from the seed of pinto bean and red bean showed numerous potential pharmaceutical applications (Table 2) in addition to their use as functional foods [113]. These peptides may contribute significantly to the nutritional value of the bean. In addition to antifungal activity, antiproliferative activity towards tumor cells including human liver hepatoma cells Bel-7402 and neuroblastoma cells SHSY5Y was reported for *Phaseolus limensis* (Table 2) [114].

PA1b (Pea Albumin 1, subunit b), a 37aa peptide first isolated from garden pea seeds, has been reported to possess strong bioinsecticidal activity against cereal weevils such as (*Sitophilus oryzae*, *S. zeamais*, *S. granarius*) or the pea aphid (*Acyrthosiphon pisum*) via induction of apoptosis through PA1b/V-ATPase interaction resulting in insect death [115]; however, this highly selective pea peptide and its analogues have not been investigated for potential therapeutic application following from the ethnopharmacological uses of their host legume plants, especially the genus *Cajanus* (pigeon peas) widely used in African and Asian ethnomedicines.

**Table 2:** Phytomedicines rich in knottins, plant source, dose and potential pharmaceutical/biotechnological applications.

^#^ Plant Source	Knottins	Pharmaceutical Application	IC_50_/EC_50_/Dose	Reference
*Hedyotis biflora* Hornem. Accepted name: *Leptopetalum biflorum* (L.) Neupane and N.Wikstr.	Hedyotide B1 (HB1), B10 and B11	Hedyotide B1 possessed strong In vitro antibacterial activity against *E. coli* and *S. salivarius*, while Hedyotide B10 and B11 were active against *S. aureus* and *S. epidermidis*.	HB1 MIC: 3.4–5.9 μM HB10 and 11: MIC 1.5–2.2 μM	[80]
*Viola odorata var*. variegata DC	Cycloviolacin O2, O3, O19	Broad spectrum antibacterial activity, CyO19 being the most potent. Cy O2 showed strong in vivo activity in surgical *S. aureus* wound infection mouse model	CyO19MIC 10 μM against *E. coli*, *P. aeruginosa* and *S. aureus*. *S. aureus* MIC 25 μM (in vivo)	[82,83]
*Oldenlandia affinis* (Roem. and Schult.) DC.	Kalata B1, B2, B7	KB1 displayed moderate activity against *P. aeruginosa*, while KB7 displayed potent activity. KB2 showed mild in vivo activity in the surgical *S. aureus* wound infection mouse model. *A. salmonicida*, *A. hydrophila*, *V. anguillarum*, *V. ordalii*, *F. psychrophilum*	KB1 MIC 40 μM KB7 MIC 1.25 μM KB2 MIC 50 μM	[82,83]
*Viola arvensis* Murray	VarcA	Antimicrobial activity against Gram-negative bacterial infection in aquaculture. (*A. salmonicida*, *A. hydrophila*, *V. anguillarum*, *V. ordalii*, *F. psychrophilum*)	MIC 12.5–30.0 μM	[86]
*Sambucus nigra* L.	-	Antimicrobial activity against Gram-negative bacteria affecting aquaculture.	100 µg/mL gave the strongest antimicrobial activity.	[25,86]
*Clitoria ternatea* L.	Cationic cT15, cT19 and cT20	Potent activity against Gram-negative bacteria (*E. coli*), antibacterial activity diminished by high salt concentration, except for cT15. Immunomodulating activity presents an opportunity to be used as an adjuvant in vaccines.	MIC 0.5–0.6 μM (low salt); cT15, cT19 and cT20 MIC 2.5 μM, >10 and >20, respectively, in high salt concentration.	
*Momordica cochinchinensis* (Lour.) Spreng.	MCoTI-II	The linear 9mer peptide grafted into the loop 6 of MCoTI-II showed reduced antibacterial activity but extreme stability in 100% human serum. Antimicrobial activity of the cyclotides was also slightly reduced in 10% human serum.	*P. aeruginosa* 0.8 and 9 for linear and cyclic peptide, respectively, in 1/5 muller–hilton *E. coli* 0.4–0.8 and 4.0–9.0 for linear and cyclic peptide, respectively, in 1/5 muller–hilton *Klebsiella pneumonia* 1.5 and 71 for linear and cyclic peptide, respectively, in 1/5 muller–Hilton.	[116]
*Hedyotis biflora* Hornem. Accepted name: *Leptopetalum biflorum* (L.) Neupane and N.Wikstr.	Hedyotide B7	Potent in both in vitro and in vivo experiments against four different pancreatic cell lines.	IC50 of 0.68, 0.45, 0.33 and 0.36 μM against BxPC3, Capan2, MOH-1 and PANC1, respectively.	[91]
*Psychotria leptothyrsa* Accepted name: *Psychotria defretesiana* W.N.Takeuchi	Psyle A, C and E	Activity on the Human Breast Adenocarcinoma Cell Line (MCF-7) and Its Drug Resistant Subline (MCF-7/ADR) similar to that of CyO2, psyle E is the most potent, and could be combined with conventional anticancer drugs to facilitate drug absorption by cancer cells.	Psyle E IC_50_ 0.64, 1.73 and 0.39 μM against MCF-7, MCF-7/ADR and co-exposure to doxorubicin, respectively.	[90]
*Hedyotis diffusa* Accepted name: *Scleromitrion diffusum* (Willd.) R.J.Wang	DC1, DC2 and DC3	Potent cytotoxic activity against human prostate cancer xenografts in both in vivo and in vitro experiments. DC3 is the most potent.	DC3 IC_50_ of 0.21, 0.76, 0.55 μM against LNcap, PC3, and DU145, respectively.	
*Melicytus latifolius* (Endl.) P.S.Green	Lysine-rich cyclotide: Mela 1–7, Mech 2 and 3.	Strong cytotoxic activity with significantly low hemolytic activity even at high peptide concentration. Their distinct toxicity properties from the usual cyclotide makes them a promising scaffold for drug design.	mela 1 CC_50_ of 2.09 ± 0.18, 3.07 ± 0.15, 9.83 ± 0.78 and >64 μM against MM96L, HFF-1, HELA and RBCs.	[93]
*Viola ignobilis* Rupr. Accepted name: *Viola odorata* subsp. *odorata*	Vigno 5	Cytotoxic against Hela cells in a dose-dependent manner.	cell viability reduced significantly in a dose dependent manner (2.5, 5.0, 7.5, and 10 μM) to 48%, 35%, 24% and 18% of total, respectively.	[94]
*Viola odorata* L. Accepted name: *Viola odorata* subsp. *odorata*	CyO2	Potent cytotoxic activity against breast cancer cell line MCF-7 and its drug resistance subline MCF-7/ADR. Selectively enhances the uptake of chemotherapeutic drug doxorubicin by cancer cells due to increased pore formation.	CyO2 IC_50_ 3.17, 3.27 and 0.76 μM against MCF-7, MCF-7/ADR and co-exposure to doxorubicin, respectively.	[90]
*Momordica cochinchinensis* (Lour.) Spreng. *Momordica cochinchinensis* and *Spinacia oleracea* L.	MCoTI-II MCoTI-Var. 4 SOTI-Var. 1	MCoTI-II scaffold has been employed in the design of knotting stabilized anticancer drugs because it lacks hemolytic activity against erythrocytes. Have high-affinity inhibitory activity against human matrypase-1 present on the surface of human prostate carcinoma cancer cells (PC-3).	- MCoTI-Var. 4 IC_50_ 0.21 ± 0.11 μM SOTI-Var. 1 IC_50_ >10 μM	[117,118,119]
*Clitoria ternatea* L.	Cliotide T1 and T4	Potent cytotoxic activity against HeLa cell line However, hemolytic activity occurs at lower doses compared to Cliotide T2 and T3.	IC_50_ 0.6 μM for Cliotide T1 and T4 HD_50_ 7.1, >100, 13.1 and 8.4 μM for Cliotide T1, T2, T3 and T4, respectively.	[80]
*Chassalia Parvifolia* K.Schum	Circulin A and B	First reported antiviral activity against HIV.	IC_50_ 500 nM and EC_50_ about 40 to 260 nM in all assays.	[99]
*Oldenlandia affinis* (Roem. and Schult.) DC.	Kalata B1, kalata B1-inspired peptide	KB1 had strong antiviral activity against HIV through disruption of viral membrane envelope. Anti-HIV activity 25 times lower than its cytotoxic concentration, very low chance of emergence of resistance. In contrast, kalata B1-inspired peptide produced from the amino acid modification of KB1 displayed significant activity against Dengue Virus.	VC_50_ of 2.04 μm for HIV-1 NL4.3 and 4.54 μm for HIV-1 Clade A No free access to the article.	[104,105,106]
*Alstonia scholaris* (L.) R.Br.	Altoids As1 and As3	Significant membrane permeability and early phase antiviral activity against infection of bronchitis virus (IBV) and Dengue infection (DENV2). Highly stable in the metabolic environment, target specificity and non-cytotoxic even at high peptide concentration made them a good candidate for drug design.	Pretreatment with As1 and As3 inhibits plaque formation in a dose-dependent manner with estimated EC_50_ of 35 and 55 μm, respectively, against IBV. EC_50_ of ∼90 μm for As1 against DENV2 NS3 protein expression in the infected cultures.	[107]
*Viola odorata* L.	CyO2	CyO2 alone decreased HIV-1 infected HuT78 cells and co-exposure increased the anti-HIV activity of Saquinavir and Nelfinavir.	Short term exposure to CyO2 (0.5 μM) alone also showed a 70–80% reduction in HIV p24 levels; while continuous co-exposure to SQV (0.004 μM) and CyO2 (0.025 μM) resulted in significant suppression (~70–80%) in HIV-1 p24 levels.	[90]
*Viola odorata* L.	CyO2, CyO3, CyO19	Potent activity against *C. albicans*	99% activity seen at Minimum Fungicidal Concentration (MFC) of 10 µM.	[82]
*Viola odorata* L.	CyO2, CyO3, CyO19, CyO8	Potent activity against fungal plant pathogens. Currently being investigated as a potential bio-fungicide against Fusarium head blight in crops	Most potent is cyO3 (MICs ranging from 0.8 to 12.5 μM), and the least active was cyO13 (MICs ranging from 3 to 25 μM).	[111,112]
*Oldenlandia affinis* (Roem. and Schult.) DC.	kalata B1 mutant [T20K]	Immunosuppressant effect is currently under clinical trial for the management of multiple sclerosis.	Single administration of T20K to healthy animals did not exhibit toxic effects up to the doses of 15 mg/kg (i.v), 75 mg/kg (i.p) and 250 mg/kg (p.o).	[20,120]
*Clitoria ternatea* L.	Cationic cliotides cT7 and cT19	Strong immunostimulating effect by enhancing the release of cytokines and chemokines.		[26]
*Viola philippica* Cav.	Kalata B1, Varv A, Viba 15, Viba 17, VarvE and Viphi G	Antibiofilm activity using *S. aureus* was reported for Viba 17, VarvE and Viphi G with Viphi G being the most potent.	At a concentration of 20 µg/mL, Viphi G inhibited bacterial growth more than 40%, while Viba 17 and Varv E only caused inhibition of 10–20%.	[121]
*Tragia benthamii* Baker	Knottin-rich biologically active peptides detected but not yet characterized.	Strong anti-inflammatory activity in vivo chick model.	Extract (30–300 mg/kg, i.p) produced a dose dependent reduction in foot edema with maximal inhibition of 84.3% at 300 mg/kg body weight.	[77]
*Ecballium elaterium* Accepted name: *Ecballium elaterium* subsp. *dioicum* (Batt.) Costich	*Ecballium elaterium* trypsin inhibitor II (EETI-II)	When engineered with scaffold proteins, were found to inhibit the binding of fibrinogen to αIIbβ3 in vitro. Engineered peptides bind to integrin expressing tumor cells. Could be employed both in the treatment and diagnosis of cancer.	Microprotein ETRGD1 with IC_50_ 4.50 ± 0.70 nM. IC_50_ 20 times lower than that of the standard drug eptifibatide. Echistatin is the most potent with IC_50_ of 4.9 ± 1.0 nM in binding assay; 9.2 ± 1.5 nM and 0.90 ± 0.2 nM cell adhesion assay using fibronectin and vitronectin, respectively.	[122,123]
*Oldenlandia affinis* (Roem. and Schult.) *DC*.	Kalata B1	In bioengineering, they become excellent scaffolds for radioactive imaging probes to be used in positron emission tomography (PET), single photon emission computed tomography (SPECT). These scaffolds have also been combined with non-radioactive probes for fluorescence and ultrasound imaging. Useful as drug delivery vehicles for bioactive therapeutic peptides.		[23,124]
*Momordica cochinchinensis* (Lour.) Spreng.	Cystine-knot peptide	This peptide has been engineered to bind to cytotoxic T Lymphocyte-associated antigen-4 (CTLA-4) which happens to be a target in the treatment of metastatic melanoma.		[125]

^#^ The accepted binomial names of listed plants have been added following information obtained from the Plants of the World Online database (https://powo.science.kew.org/ database) (accessed on 27 April 2022).

## 3. Limitations to the Pharmaceutical Applications of Knottin-Rich Peptides in Clinical Settings

**Figure 5:**
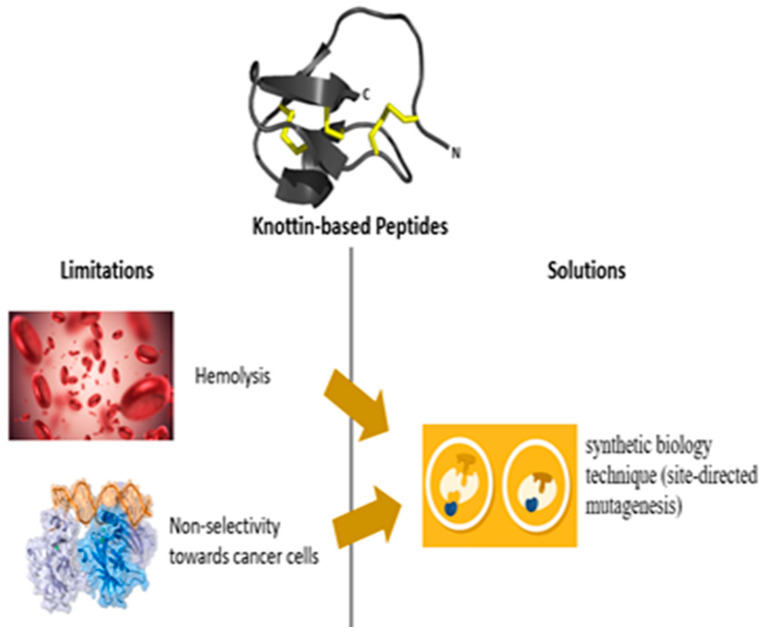
Highlights of some limitations and proposed solutions in enhancing the use of Knottins in clinical therapy.

Knottin-rich peptides and phytomedicines rich in knottins have demonstrated numerous applications and clinical uses that range from insecticidal, cytotoxic, antimicrobial, antiviral, antineoplastic and much more [17], yet limitations to their adoption abound (Figure 5). These limitations include unwanted properties, characteristics or effects that are clinically undesired. The broad range of activity of some of these peptides, however important, may sometimes lead to unwanted effects. One of such effects is hemolysis. Though some knottins, such as plant defensins, have been reported to be non-toxic to human cells [126], a few cyclotides have shown hemolytic potential to mammalian erythrocytes [81]. This ability to lyse red blood cells occurs via its interaction with membranes—the same mechanism by which it kills microbes. So far, it has been observed that the level of hemolytic activity varies from peptide to peptide and often correlates with parasiticidal activity [127]. Cycloviolacin H4, Cycloviolacin Y5, VarvE, characterized from *Viola hederacea*, *Viola yedoensis*, and *Viola tricolor* are examples of peptides which have shown hemolytic activity [128]. As expected, this hemolytic effect limits the applications of some knottin peptides (particularly cyclotides) in clinical therapy, but fortunately knottins are easily modified and eliminating this unwanted property using a synthetic biology technique known as site-directed mutagenesis is achievable. This will be crucial in enhancing its therapeutic suitability and wider adoption.

Generally, knottins such as cyclotides are known to be target specific. However, the usefulness of some knottins as cytotoxic agents in the treatment of cancer may be limited in its clinical application due to target non-selectivity. Some of the cytotoxic knottins affect both healthy and unhealthy cells. This is a major problem faced in cancer therapy. The majority of existing cancer treatment options are faced with this drawback which reduces beneficial outcomes for patients. The cytotoxic effect is also related to their ability to bind to cell membranes. On the bright side, recent studies have reported a higher selectivity among some cyclotides [94]. Another potential limitation in clinical application is low immunogenicity. Some cyclotides were discovered to be immunologically unresponsive without antigen conjugation [129]. Although these prospective clinical uses have piqued interest in cyclotides, a better understanding of their immunological characteristics is required and demands closer scientific attention.

Many knottin peptides are still in pre-clinical trials, but Table 3 highlights knottin peptides that have made it to clinical trials. Prominent among them is the nature-inspired T20K kalata B1, originally characterized from the African traditional plant *Oldenlandia affinis*. This knottin-like cyclotide has demonstrated therapeutic promise in inflammatory-related and autoimmune disorders [13,120]. A linear knottin, bevuTI-I, isolated from the aqueous extracts of *Beta vulgaris*, showed interesting mammalian enzyme inhibitory activity against trypsin (IC_50_ = 471 nM) and human prolyl oligopeptidase (IC_50_ = 11 μM), a drug target for neurodegenerative and inflammatory disorders [13]. It is of note that the same aqueous extracts of this plant are currently under clinical investigation for blood pressure, inflammatory markers and oxidative stress in type 2 diabetes (Table 3). Knottins such as cyclotides and their linear variants are well reported as highly specific and stable peptide-based protease inhibitors. The documented inhibition of prolyl oligopeptidase by knottin-like cyclotides [13,130] should trigger scientific investigation of the potential of knottin-like peptides, to inhibit the enzymes involved in a cascade of reactions leading to the extracellular plaques of beta-amyloid peptide (Aβ) deposits in the brain observed in Alzheimer’s disease [131]. As a first step, Kalmankar and colleagues [132] showed that cyclotides from *Clitoria ternatea* can inhibit the aggregation of amyloid β peptide (Aβ). This computational study showed that cyclotides can prevent Alzheimer’s progression by binding to peptide structures and inhibiting their aggregation. Following a thorough molecular dynamics simulation analysis, it was discovered that cyclotides reduce the inter-strand hydrogen bonds between the Aβ peptide by forming hydrogen bonds with the hazardous amyloid assemblies. While there is a dearth of clinical trials on the use of knottin-like peptides in Alzheimer’s disease, this computational study suggests its possible potential in Alzheimer’s therapy and highlights the need for further research. Other knottin peptide-rich phytomedicines that are in clinical trials for at least one human disease include Zufa (containing *Viola odorata*), *Alstonia scholaris*, *Moringa oleifera* and *Momordica cochinchinensis* (Table 3).

**Table 3:** Phytomedicines rich in knottin peptides and purified Knottin peptides from Botanical sources in clinical trials. Many of the observed clinical activities presented in the table have been attributed to other secondary compounds, while bioactive knottins present in the aqueous/alcoholic extracts have not been considered. This represents a huge gap for peptide drug discovery, warranting further exploration.

Knottins/Phytomedicine	Source	Clinical Trial	IC_50_/Dose	Reference
T20K	*Oldenlandia affinis*	Multiple sclerosis	2.7 ± 0.62 µM S.D	[120]
Integrin αvβ6 PET tracers (R_0_1-MG)	Engineered peptides	imaging	0.56 ± 0.46 nM S.D	[122]
Herbal Cyclotide Complex syrup	Cyclopeptide fraction with *Ziziphus spina-cristi* and *Pimpinella anisum hydroalcoholic* extract and orange peel	Post-exposure prevention of infection with COVID-19.	Dose = 20 mL every 8 h for 14 days	Clinical Trial Protocol Iranian Registry of Clinical Trials (https://www.irct.ir/trial/49925, accessed on 27 April 2022)
Concentrated Beet Root Juice	Knottin peptide-rich juice of the root of *Beta vulgaris*	Aortic and brachial blood pressure over 24 h	70 mL concentrated beetroot juice drink	[133]
Concentrated Beet Root Juice	Knottin peptide-rich juice of the root of *Beta vulgaris*	Inflammatory markers and oxidative stress in patients with type 2 diabetes	12 mL concentrated beetroot juice twice daily	[134]
Zufa syrup (Cyclotide-rich Polyherbal extract)	Herbal mixture containing three plants including the cyclotide-rich *Viola odorata*	symptomatic benign prostate hyperplasia (BPH) men	7.5 mL of syrup every 4 h for 10 days	[135]
Knottin peptide-rich Gac extract	*Momordica cochinchinensis*	antiwrinkle agent	5% Gac extract in cream applied to the entire face twice daily	[136]
Knottin peptide-rich Alkaloid extract	*Alstonia scholaris*	bronchitis, post-infectious cough and asthma.	8–480 mg, three times daily for 7 days	[137]
Flower extract of *Clitoria ternatea*	*Clitoria ternatea*	glycemic response and antioxidant capacity	1 g–2 g in 400 mL of water	[138]
Knottin peptide-rich *Moringa oleifera* leaf powder divided twice daily in corn porridge *Moringa oleifera* leaf powder	*Moringa oleifera*	maternal and infant healthsupplementation on the immune status and anthropometric parameters of adult HIV patients	20 g daily for 3 months15 g Moringa leaf powder sachets (5 g each for 3 times)	[139,140]

## 4. Materials and Methods

The many prominent academic databases including Scopus, Google Scholar, Pubmed, Researchgate, Science Direct, Medline, Embase and Cochrane were searched in order to retrieve relevant data on knottin peptides. Search phrases fed into the databases include “knottins”, “knottin peptides”, “cyclotides”, “plant defensins”, “hevein-like peptides”, “cystine knot peptides”, “cysteine-rich peptides”, “bioactivity”, “pharmaceutical application”, “nutritional application”, “anticancer and antiviral and antibacterial”, “benefits” and “applications”. Focused attention was given to quality, peer-reviewed papers published within the last 10 years (2013–2022).

Findings from keywords were downloaded and archived. Papers excluded from the list are those which do not meet the inclusion criteria for knottins: molecular weight of 2–6 kDa, scientifically sound and phytomedicines rich in knottins occupying the interphase of food and medicine. Knottin peptides from animal sources were excluded.

## 5. Conclusions and Prospects

Knottin peptides, biosynthesized in several edible botanical sources, have been poorly investigated for their benefits, bioactivity and nutritive properties. In the future, knottins with validated, acceptable levels of safety should be investigated for use in creating functional food products with an extended shelf life; this is already evident by their reported property as antioxidants in fatty foods and as anti-infective/antimicrobial agents in food packaging. In-depth and elaborate pre-clinical and clinical research is needed to discover which knottin peptides are favorable for clinical use, their dose/response relationship, absorption, pharmacokinetic and compatibility with various foods.

Knottins are peptide-based phytoconstituents whose contributory nutritional/pharmacological roles are largely unknown in many standardized phytomedicines. The therapeutic and nutritional roles of knottin peptide components of these phytomedicines have not received the attention of the scientific community despite their amazing potential in the food–medicine space. Thus, one of the future prospects in the use of knottins may be to investigate their potential as alternative dietary protein source and functional amino acids that could produce a therapeutic benefit at the highest level of clinical evidence.

Future prospects for the effect of knottin in antimicrobial studies would be to test many more and establish their activities against multi-resistant strains of various microorganisms as well as their clinical isolates. It would also be important to establish their mechanism of actions in executing these antimicrobial activities and justify their safety for inclusion as functional food additives.

Knottins are also enriched with excellent protein scaffolds that can be explored in the search for new drug entities in cancer drug management and drug delivery. They can equally serve as a carrier in drug delivery, especially in overcoming the blood–brain barrier which could be difficult for several bioactive conventional drug candidates.

Members of the knottin peptides are amenable to molecular imaging applications owing to their rapid renal clearance. This property is relevant to pharmacokinetic studies and could fast track novel peptide drug discovery for the management of the most challenging metabolic diseases. Knottin peptides therefore seem to fulfill the 21st-century requirement as non-invasive diagnostic agents awaiting beneficial exploitation.

In conclusion, reported knottin peptides are mainly gene encoded making them suitable candidates for engineering applications. This increases their potential for numerous therapeutic and nutraceutical applications via synthetic biology approaches and the precision tool of site-directed mutagenesis. Although the database for knottin peptide-based molecules is continuously growing, more elaborate research on knottins is highly encouraged to expand the database and explore novel aspects of their nutraceutical and therapeutic applications.

## Data Availability

Not applicable.
